# Estimating cumulative risks for breast cancer for carriers of variants in uncommon genes

**DOI:** 10.1007/s10689-016-9896-2

**Published:** 2016-03-09

**Authors:** Noralane M. Lindor, John Hopper, James Dowty

**Affiliations:** Department of Health Sciences Research, Mayo Clinic, 13400 East Shea Blvd., Scottsdale, AZ USA; Melbourne School of Population and Global Health, University of Melbourne, Parkville, VIC 3010 Australia

**Keywords:** Penetrance, Hazard ratios, Odds ratios, Hereditary breast cancer, Nextgen, Cancer panels

## Abstract

The rapid clinical embrace of next generation multigene cancer predisposition panels has resulted in discovery of DNA variants in genes for which very limited data on penetrance has been published. Evidence for increased risks associated with these genes is often expressed in odds ratios and studies often were conducted on a priori high risk cohorts, i.e. those with young onset disease and/or positive family histories. Despite these limitations, one can estimate cumulative risks, which may be useful for health care providers who are counselling individuals on their results. We present cumulative risks for several under-studied genes and provide generic information that can be extrapolated to data still emerging.

## Introduction

For clinicians providing cancer risk assessment for carriers of rare variants in genes for which there is very limited information on risk, one problem is the non-uniform manner in which risk estimates are presented. Cumulative lifetime risk, the most familiar and intuitive concept, is typically used in clinical guidelines such as the US Preventive Services Task Force recommendation to offer magnetic resonance imaging screening to women whose lifetime risk of breast cancer exceeds 20–25 %. Therefore, when publications present odds ratios (ORs), relative risks, or standardized incidence ratios, it is necessary to convert those estimates.

## Methods

Following a literature review, we compiled examples of published risk estimates for pathogenic variants now being detected by some of the new breast cancer gene panels and have converted these so the risks for carriers can be expressed in terms of cumulative risks (Table 1). To calculate the cumulative risks, we used USA population incidences compiled by the International Agency for Research on Cancer and a formula relating a hazard ratio to the age-specific cumulative risks. In detail, the age-specific cumulative risk to age T years for carriers was calculated as one minus the exponential of minus the cumulative incidence to age T years, where the cumulative incidence to age T years is the sum, as S ranges over all ages between 0 and T years, of the population incidence at age S years times the hazard ratio at age S years. We assumed that the hazard ratios (definition: the ratio of age-specific breast cancer incidence in carriers to that in non-carriers) for the genetic variants are approximately equal to their reported ORs (definition: the ratio of the odds of breast cancer for carriers divided by that of non-carriers), standardized incidence ratios (definition: ratio of the observed rate of breast cancer to the age-adjusted expected rate in the general population) or relative risks (definition: the ratio of the risk of breast cancer in gene carriers compared to the risk among those who are not gene carriers).

**Table 1:** Gene penetrance as reported in selected publications for some breast cancer predisposition genes

Gene	Study design	Ethnicity	No. cases	No. controls	OR; RR	95 % CI	Percentage cumulative risk to age 70 years for carriers living in USA (95 % CI)	Comment	References
BLM c.1642C>T (Q548X)	Case: Control	Russian	1498	1093	OR 6.28	1.52–42.18	43 (12.7–97.7)	Some selection for high risk; OR calculated using only unselected cases (n = 879). Mean age for cases, 51 years; range 24–81 years. Controls were cancer-free blood donors	[2]
BRIP1 truncating mutations	Case: Control	United Kingdom	2012	2081	RR 2.0	1.2–3.2	16.4 (10.2–24.9)	*BRCA1/2* negative women with breast cancer and family history of breast and/or ovarian cancer. Relative risks in whole cohort. No ages provided. Controls from a 1958 birth cohort collection, not selected for any other characteristic	[3]
BRIP1 truncating mutations	Case: Control	United Kingdom	Unknown	2081	RR 3.5	1.9–5.7	26.9 (15.6–40)	Subset of cohort of women with breast cancer and family history of breast and/or ovarian cancer. Relative risk for carriers < 50. Controls from a 1958 birth cohort collection, not selected for any other characteristic	[3]
NBN 657del5	Meta-analysis of 9 case:control studies	Multinational	7534	14,034	OR 2.63	1.76–3.93	21 (14.6–29.7)	Meta-analysis of 9 studies	[4]
NBN 657del5	Case: Control	Polish	66	1620	OR 8.36	2.57–27.27	52.7 (20.6–91.3)	Cases diagnosed <40 years. Controls were anonymous donors, not otherwise described	[5]
NBN 657del5	Case: Control	Polish	221	1620	OR 4.27	1.67–10.89	31.8 (13.9–62.3)	Cases 40–50 years. Controls were anonymous donors, not otherwise described	[5]
NBN 657del5	Case: Control	Polish	341	1620	OR 2.4	0.91–6.35	19.3 (7.8–43.4)	Cases ≥ 50 years. Controls were anonymous donors, not otherwise described	[5]
NBN 657del5	Case: Control	Polish	562	1620	OR 3.13	1.4–7.00	24.4 (11.8–46.6)	All (unselected) cases. Age range 24–81 years; median 52 years	[5]
RAD50 687delT	Case: Control	Finnish	317	1000	OR 4.3	1.5–12.5	32 (12.6–67.3)	Unselected cases. Mean age of cancer, 58 years; range 29–95 years. Controls were anonymous cancer-free blood donors	[6]
RECQL c.1667_1667 + 3delACTA	Case: Control	Polish	13,136	4702	OR 5.4	1.3–46	38.3 (11.0–98.4)	Unselected cases. Age ranges not provided. Controls not selected for cancer or family history	[7]

Given the discordance of risk estimates across studies, caution is warranted in clinical interpretation (see “Discussion”). Additional new studies and estimates are emerging rapidly for specific genes

*CI* confidence interval, *OR* odds ratio, *RR* relative risk

## Results

Table 1 shows the published risk estimates for specific genes and/or variants within genes. Using the strategy described, this risk is converted to a cumulative risk to age 70 years for carriers living in the USA and other countries with similar population incidences.

## Discussion

Establishing penetrance is a difficult proposition, even for extensively studied genes, like *BRCA1* and *BRCA2*, in which no two studies have ever yielded the same results. Discrepancies are attributable to differences between populations and to methodology. The range of penetrance generally cited for the *BRCA* genes drives the clinical recommendations for surveillance and prevention, and provides a yardstick by which other genes can be compared and contrasted. Conducting comparisons is very difficult when the only published literature does not provide cumulative risks/penetrance for these new genes. In this paper, we sought to demonstrate one way to address this gap.

Some cautions are warranted in accepting the risk estimates provided in Table 1. Some of the published ORs were estimated using cases selected for a family history, so the corresponding ORs are only appropriate for women with comparable family histories, but the published ORs from studies unselected for family history are appropriate for all carriers in general in the population studied. In this case, the risk for an individual carrier is the product of her risk due to the variant and her risk due to any family history that she has. Note that, unlike high-risk mutations, variants associated with a moderately increased risk explain only a small portion of the cancer family history of the carriers. Therefore, for carriers with a family history, their cumulative risk will depend on the increased risks associated with both their cancer family history and the variant itself [1]. For example, if a carrier of a variant associated with an increased OR of cancer also has a family history sufficient to triple her risk, her cumulative risk will be about that of a woman with a 3 × OR increased risk.

**Fig. 1:**
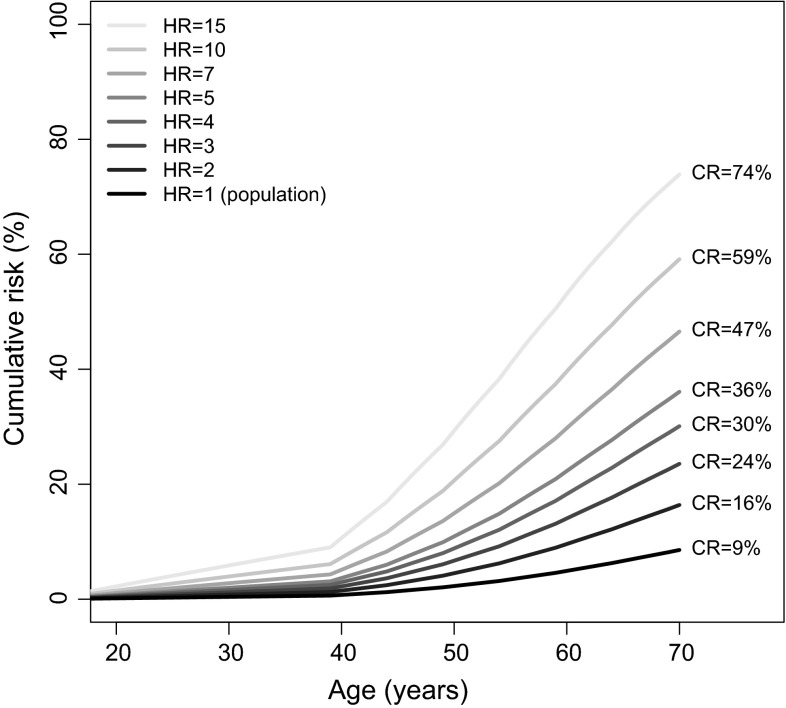
Cumulative risks (CRs) for carriers of variants with various hazard ratios (HRs), for carriers living in the USA and other countries with similar population incidences. This figure provides a general guidance for clinicians considering how risks of genes, based on very limited data yet, compare with genes with more established management strategies

In Fig. 1, we have plotted age-specific cumulative risks for breast cancer corresponding to various hypothetical hazard ratios. This generic plot can therefore be used to estimate a carrier’s overall risk including cancer family history and other known risk factors. Figure 1 can also be used to gauge the degree of uncertainty in the overall cumulative risk estimate from the uncertainty (as expressed in confidence intervals) in the risk estimates.

Additional caution is warranted in use of these estimates, because: validation studies have not been published for most of these variants so the published ORs might be subject to publication bias. The risks shown in Table 1 do not reflect all known papers on the subject and new papers are being published rapidly now; risk estimates might be population-specific; and risk estimates have come from a mixture of studies, some of which did not select cases on the basis of family history and some that did. Also, in the absence of a precise formula converting ORs to cumulative risks, we have had to assume that reported ORs are close to, if not identical to, the hazard ratios. We have also assumed that the hazard ratio is constant with age, which is unknown for these variants. For high-risk variants, such as mutations in *BRCA1*, this is not true as the population of higher risk carriers is depleted across the decades by death from breast and ovarian cancers. For less lethal variants, however, this will not be a major issue. We also assumed that the incidences for non-carriers are the same as the population incidence; this is likely true given these variants are rare.

Risk estimates are but one factor that needs to be integrated with other complex issues in individualizing medical management. We hope that the estimates and confidence intervals in Table 1, and the cumulative risks in Fig. 1, will be useful to clinicians given that cancer family history and other risk factors are appropriately taken into account.
